# Vitamin D Supplementation and Non-Alcoholic Fatty Liver Disease: Present and Future

**DOI:** 10.3390/nu9091015

**Published:** 2017-09-14

**Authors:** Ilaria Barchetta, Flavia Agata Cimini, Maria Gisella Cavallo

**Affiliations:** Department of Experimental Medicine, Section of Medical Pathophysiology, Food Science and Endocrinology, Sapienza University of Rome, Rome 00161, Italy; ilaria.barchetta@uniroma1.it (I.B.); flaviaagata.cimini@uniroma1.it (F.A.C.)

**Keywords:** vitamin D, NAFLD, therapy, nutraceuticals

## Abstract

Non-alcoholic fatty liver disease (NAFLD) is the most common chronic hepatic disease throughout the Western world and is recognized as the main cause of cryptogenic cirrhosis; however, the identification of an effective therapy for NAFLD is still a major challenge. Vitamin D deficiency is a wide-spread condition which reaches epidemic proportions in industrialized countries, mainly in relation to current lifestyle and limited dietary sources. Epidemiological studies point towards an association between hypovitaminosis D and the presence of NAFLD and steatohepatitis (NASH), independently of confounders such as obesity and insulin resistance. Furthermore, several pieces of experimental data have shown the anti-fibrotic, anti-inflammatory and insulin-sensitizing properties exerted by vitamin D on hepatic cells. However, results from trials evaluating the effects of oral vitamin D supplementation on liver damage in NAFLD and NASH are controversial. The aim of this review is to give an overview of the evidence currently available from clinical trials and to discuss possible shortcomings and new strategies to be considered in future investigations.

## 1. Introduction

Non-alcoholic fatty liver disease (NAFLD) is a pathological condition characterized by aberrant triglycerides accumulating in the hepatocytes, in some cases accompanied by necro-inflammatory activity and fibrosis (steatohepatitis) and potentially evolving into liver cirrhosis. NAFLD represents the most common chronic hepatopathy worldwide, reaching a prevalence of above 70% in patients with type 2 diabetes (T2D), obesity and metabolic syndrome [1,2]. In these groups, NAFLD may significantly worsen metabolic outcomes and, in the general population, is now considered an independent risk factor for cardiovascular disease and a major public health issue [3,4]. However, beside lifestyle intervention, no established therapy of NAFLD has been identified yet [5,6].

Vitamin D is a hormone exerting several beneficial effects beyond its role in bone homeostasis; active vitamin D has been shown to modulate the immune system, inducing an anti-inflammatory and anti-fibrogenic pattern in the liver [7,8,9,10]. Under experimental conditions, vitamin D has been shown to significantly inhibit the hepatic expression of pro-fibrotic mediators such as platelet-derived growth factor (PDGF) and transforming growth factor β (TGF-β) [7,9] and to suppress the expression of collagen, α-smooth muscle actin and tissue inhibitors of metalloproteinase-1; however, its protective action against fibrosis was ineffective once overt hepatic cirrhosis was established [9]. Furthermore, vitamin D has been proposed as an effective modulator of insulin sensitivity in several experimental models [11,12,13] and epidemiological data show the existence of a tight correlation between low circulating vitamin D levels and the presence of obesity [14], T2D [15] and insulin resistance-related conditions [16,17].

Furthermore, data from meta-analyses point towards the existence of an association between low circulating vitamin D levels and NAFLD [18]. Of note, low serum vitamin D concentration correlates with the presence and severity of liver steatosis and necro-inflammatory damage over the course of NASH in both children [19,20] and adults [21,22,23].

## 2. Clinical Trials

Based on experimental evidence and epidemiological data, vitamin D has been proposed as a potential therapeutic option for liver damage in NAFLD and non-alcoholic steatohepatitis (NASH) [24].

However, most studies aiming to test the efficacy of high dose vitamin D supplementation on NAFLD did not obtain any improvement in either fatty liver content, histological parameters related to NASH, or transaminases [25,26]. The first pilot study on NASH was conducted by Kitson MT et al. [27] in several patients undergoing liver biopsy before and after six-month 25,000 IU weekly cholecalciferol supplementation and showed no effect on liver outcomes. In this investigation, study participants had an overt diagnosis of steatohepatitis, and established inflammation and fibrosis were detected in the liver biopsy. In this condition, vitamin D failed to demonstrate any effect in reducing local inflammation and fibrosis or, at least, impacting intrahepatocyte fat accumulation [27].

Conversely, the only randomized controlled trial (RCT) testing the safety and efficacy of oral vitamin D supplementation on liver damage in children affected by biopsy-proven NASH demonstrated that the combination of daily cholecalciferol 800 IU and docosahexanoic acid (DHA) 500 mg improved the NAFLD Activity Score from baseline to end of treatment in the treated arm. The improvement trend in fibrosis score, and ameliorated parameters relating to insulin-resistance and dysmetabolism were noted as non-significant [28]. However, the comparison with trials testing the effects of DHA treatment alone on NAFLD/NASH showed similar results [29,30], suggesting that such improvements were likely related to the effect of DHA, rather than to the vitamin D treatment.

In light of these results, it could be hypothesized that the lack of favorable effects of vitamin D on NASH may partially be explained by the presence—along with an increased hepatic fat fraction—of marked fibrotic damage and chronic inflammation, as also suggested by experimental models [9]. In this scenario, the insulin-sensitizing and anti-inflammatory properties exerted by vitamin D in experimental conditions, therefore, could not restore the advanced liver injury over the course of NASH.

Soon after, we published our data on the first RCT evaluating whether high dose oral vitamin D supplementation could reduce hepatic fat accumulation in subclinical NAFLD, i.e., in patients with hepatic steatosis but normal transaminases, liver function and no indication of liver biopsy [31]. Similar to what was observed in NASH, the 24-week oral vitamin D_3_ (cholecalciferol) supplementation with 2000 IU a day provided in our study did not succeed in reducing MRI-assessed hepatic fat content in patients with T2D and NAFLD. Moreover, no beneficial effect was reported for any metabolic parameter, such as body adiposity, glycemic control, insulin resistance, blood pressure or endothelial dysfunction. Of note, almost all participants reached sufficient or optimal vitamin D levels during the first three months, representing the functional half-life interval of vitamin D_3_ within the body [32], and kept them until the end of the study.

Other investigations, with different study design and outcome measures, have been conducted to unravel possible therapeutic effects of vitamin D on hepatic damage, showing contrasting results on non-specific markers of hepatic injuries, such as blood transaminases [33,34].

## 3. Vitamin D Supplementation: A Matter of Dosing Regimen?

Very recently, Luger M. et al. [35] conducted a RCT aiming to test the efficacy and safety of different oral vitamin D supplementation schedules and demonstrated that a forced vitamin D dosing regimen, consisting of up to three oral megadoses (100,000 IU/each) in the first month after bariatric surgery, was more effective than conventional daily supplementation in restoring normal/optimal vitamin D balance and allowed the achievement of significantly higher vitamin D levels in obese patients with advanced liver fibrosis compared to subjects without hepatic fibrosis. The loading dose regimen with vitamin D_3_ was the only regimen effective in increasing 25(OH) vitamin D concentrations in patients with significant liver fibrosis over the 6-month study period in contrast to the conventional regimen using the same maintenance dose. Besides, no data on possible changes of liver parameters related to NAFLD or NASH, such as histology, serum transaminases, CK-18 and Fatty Liver Index, before and after study treatment, are available.

Interestingly, the findings by Luger M. et al. [35] are consistent with data from a non-controlled study performed on NAFLD subjects, which demonstrated reduced controlled attenuation parameters (CAP) at the transient elastography after a one-week forced vitamin D dosing regimen followed by three weeks of conventional oral vitamin D supplementation [36]. Indeed, further reduction of CAP after the first four weeks—throughout the following five months of conventional daily vitamin D regimen—was not significant [36].

Despite the obvious limitations of studies conducted in very specific populations—i.e., morbidly obese individuals undergoing bariatric surgery [35]—or which lacked a control group [36], these results give an intriguing perspective on the reason why trials so far have failed to demonstrate relevant beneficial effects of vitamin D supplementation on fatty liver and signatures of steatohepatitis.

As magisterially pointed out by Heaney [37], a central bias behind the inconsistent results from trials on the effects of vitamin D supplementation may be represented by the well-known physiology of nutrients, which do not show the dose–response relation observed for most drugs. Indeed, what should first be considered is the vitamin D balance at the baseline, since the same supplementation schedule could not produce the same results in individuals with different circulating vitamin D levels at the beginning of the study. Systemic vitamin D status is estimated by measuring the circulating concentrations of 25(OH) vitamin D, the most stable precursor of vitamin D. However, a large number of studies were conducted before the first isolation of 25(OH) vitamin D, with obvious detrimental consequences on data interpretation. In this respect, the standardization of procedures for vitamin D measurement represents a central goal for properly defining the vitamin D status and, if required, designing the most appropriate supplementation strategy [38].

Nevertheless, another important issue involves the significant inter-assays variability observed for 25(OH) vitamin D measurement [39,40]. Moreover, although commercial immunoassays—such as DiaSorin Liaison and IDS-EIA and IDS-radioimmunoassay are commonly used, Liquid Chromatography-Tandem Mass Spectrometry is now considered by some authors as the gold standard method for 25(OH) measurement [41].

Therefore, such a lack of common standards may represent an important limit for the interpretation of individual 25(OH) vitamin D concentration measurements in a clinical setting, for the identification of standardized cut-offs for defining hypovitaminosis D, and, mostly, for comparing research findings on vitamin D status assessed by different methods.

Second, and perhaps most important, once an optimal balance of nutrients has been restored, no additional effects can be produced.

Beside the clinical trials performed in patients with NAFLD evaluating the safety and efficacy of vitamin D supplementation on parameters related to liver steatosis and hepatic damage, several studies, performed in different populations, have shown the superiority of vitamin D loading dose in comparison with oral daily vitamin D supplementation in restoring 25(OH) vitamin D levels. Wijnen H. et al. [42] demonstrated that the percentage of nursing home patients reaching optimal vitamin D concentrations after a 26-week treatment was significantly higher among individuals undergoing vitamin D loading in comparison with those treated with oral daily vitamin D supplementation (83% vs. 13%). In this study, the loading dose was calculated by an algorithm taking into account both basal and target 25(OH) vitamin D levels and body weight and was administered in separate doses of 50,000 IU twice a week until the total loading dose was reached [42].

Similar results have been obtained in pediatric populations [43]. Data from a systematic review aiming to identify the most favorable vitamin D dosing regimen for restoring normal 25(OH) vitamin D levels in children, shows that the administration of a loading dose of vitamin D which considers current vitamin D status, baseline 25(OH) vitamin D concentration and age or weight (10,000 IU/kg; maximum: 400,000 IU)—instead of daily supplementation—represents the most appropriate regimen for rapidly elevating circulating 25(OH) vitamin D concentrations [43].

High-dose loading vitamin D regimen (cholecalciferol 300,000 IU) was demonstrated to be superior to daily treatment in correcting hypovitaminosis D and normalizing PTH suppression in patients affected by rheumatic diseases, such as autoimmune/inflammatory rheumatic disease and osteoarthritis [44].

Recently, results from the REVITAHIP—Replenishment of Vitamin D in Hip Fracture strategy-trial [45] showed that high-dose vitamin D loading with cholecalciferol 250,000 IU, followed by daily vitamin D and calcium supplementation for 26 weeks was safe and more effective in improving 25(OH) vitamin D concentrations, reducing falls and pain levels, than placebo and 26-week daily vitamin D and calcium supplementation in old patients that underwent hip fracture surgery, although the two study arms reached comparable circulating 25(OH) vitamin D levels at the end of the study.

Therefore, a loading dose capable of filling the central distribution volume for vitamin D to a concentration which matches the final plateau concentration achieved with the maintenance dose, may display several advantages: forced vitamin D dosing regimens have been shown to be safe [35]; reaching optimal vitamin D balance sooner exposes individuals to potentially beneficial levels for a longer time, with additional chances to obtain favorable effects from the supplementation; the vitamin D load may somehow overcome some of the detrimental processes shown over the course of NAFLD and, mostly, NASH.

## 4. Other Factors Influencing Vitamin D Dose–Response

Along with baseline circulating 25(OH) vitamin D levels and dosing regimens, there are other factors which may display a critical role in determining the overall vitamin D balance and, thus, its therapeutic effects on organs and systems [46,47,48,49,50,51,52,53,54,55,56,57].

First, it is important to consider vitamin D biology and inter-individual differences in its metabolism. Once produced in the skin surface through an UV-mediated reaction, ingested with the food or therapeutically administrated by different routes, vitamin D is mainly stored in the adipocytes and, therefore, its bioavailability is influenced by the total adipose tissue volume [47]. Subsequently, as largely demonstrated, obese individuals generally have lower circulating vitamin D levels than lean subjects, his difference is even more marked in presence of metabolic syndrome [17] and need larger vitamin D amounts for restoring a normal balance when undergoing vitamin D supplementation [48].

In addition, genetic factors exert a significant influence on both baseline vitamin D levels and individual responses to vitamin D supplementation [49,50,51,52,53,54].

Recently, Zhang M. et al. [49] in an extensive study conducted on over 2200 postmenopausal women, investigated the association between 291 polymorphisms and circulating serum 25(OH) vitamin D levels before and after vitamin D and calcium supplementation and demonstrated that the vitamin D-related CYP2R1 and GC genes—the latter encoding for Vitamin D-binding protein, VDBP—contribute to the variability of baseline vitamin D concentration, whereas the rs11185644 polymorphism, sited near the retinoid X receptor α (RXR α) gene, influences 25(OH) vitamin D dose–response variations independently of age, body mass index (BMI) and seasonality [49].

Genetics may also explain the different prevalence of hypovitaminosis D registered among different ethnicities [50]. It is known that circulating vitamin D levels are commonly lower in black than in white Americans, although the latter have a higher incidence rate of bone frailty and fractures [51]. VDBP is the main vitamin D carrier protein, binding 85 to 90% of total circulating 25(OH) vitamin D, and inhibits some vitamin D action since the bound fraction may be unavailable to act on target cells [52]. Thus, genotyping studies demonstrated that two VDBP polymorphisms—rs4588 and rs7041—highly prevalent in general population and associated with the race, are not only associated with serum 25(OH) vitamin D levels [49,50] but also explain almost 80% of circulating VDBP inter-individual variation. Therefore, by reducing both VDBP and 25(OH) vitamin D, VDBP-associated SNPs may justify equal vitamin D availability and action in different ethnic groups, independent of measured 25(OH) vitamin D concentration [50]. Not secondarily, these data point out the importance of evaluating VDBP levels in the determination of overall vitamin D balance.

Vitamin D Receptor (VDR) polymorphisms have also been associated with inter-individual responses to vitamin D treatment, in terms of both serum 25(OH) vitamin D increase [53,54] and modification of metabolic parameters, such as insulin-resistance, glycosylated hemoglobin and blood lipid profile, after vitamin D_3_ supplementation [54].

Epigenetics, and, in particular, differential DNA methylation, is considered as another possible determinant of dose–response concentration after vitamin D supplementation. Zhou Y. et al. [55] showed that baseline DNA methylation levels of CYP2R1 and CYP24A1, two of the cytochromes involved in vitamin D metabolism in humans, were able to predict vitamin D response variation in a population of postmenopausal women.

Finally, another significant aspect to consider is the therapeutic approach to adopt when setting a therapy with vitamin D. Several vitamin D metabolites are currently available as different strategies of supplementation for restoring normal vitamin D levels; among them, the main contenders are represented by vitamin D_3_ and vitamin D_2_ (ergocalciferol). Meta-analyses and guidelines currently indicate cholecalciferol as a first-choice treatment in relation to its superiority in raising 25(OH) vitamin D in comparison with ergocalciferol, mostly when used in a single large-dose regimen [56,57]. The action of vitamin D downstream metabolites, such as 25(OH) vitamin D and 125(OH)_2_ vitamin D, has also been largely investigated, and evidence has shown an increased risk of hypercalcaemia and hypercalciuria associated with these therapies [58].

## 5. Conclusions

Several experimental studies point towards a direct role of vitamin D in modulating liver inflammation and fibrogenesis and improving hepatic response to insulin, likely through the binding to specific VDR expressed on different cell types into the liver [7,59,60].

Along with these data, the epidemiological evidence of an association between hypovitaminosis D and the presence of NAFLD [22,23,61] reinforces the rationale behind an intervention aiming to restore optimal vitamin D levels as a therapeutic option for the management of NAFLD. In the light of these considerations and critically examining results from trials conducted so far, it may be reasonable to reconsider whether there could still be room for further investigations on vitamin D supplementation in NAFLD and NASH.

Vitamin D is a molecule exerting beneficial effects in several biological systems, besides its central role in bone and mineral homeostasis. Furthermore, the measurement of blood vitamin D concentration is validated and now standardized and, therefore, allows to both personalize the supplementation regimen in relation to levels at baseline, and monitor the safety of the treatment throughout the intervention period. The overall number of RCTs investigating the effects of vitamin D supplementation in NAFLD and NASH is very limited and some interesting input came from recent trials with vitamin D dosing regimens that differ from the ones tested before. Further investigations with appropriate study design may be needed before drawing final conclusions on the benefit of restoring optimal vitamin D balance on the hepatic damage in fatty liver diseases.
